# Focal Seizures in a Child Following COVID-19 Infection: A Case Report

**DOI:** 10.7759/cureus.22083

**Published:** 2022-02-10

**Authors:** Janardhan Mydam, Srinivas Midivelly, Pujitha Vallivedu Chennakesavulu, Arnav Mydam, Hundana Allepalli, Kiran Depala

**Affiliations:** 1 Neonatology, John H. Stroger Jr. Hospital of Cook County, Chicago, USA; 2 Pediatrics and Child Health, Yashoda Hospital, Hyderabad, IND; 3 Internal Medicine, Kamineni Academy of Medical Sciences and Research Centre, Hydearbad, IND; 4 Research, Berkeley Preparatory School, Tampa, USA; 5 Research, Adlai E. Stevenson High School, Buffalo Grove, USA; 6 Research, College of Public Health and Social Justice, Saint Louis University, St. Louis, USA

**Keywords:** methyl-prednisolone, intravenous immunoglobulins (ivig), case report, neurology, child health, focal seizures, covid-19

## Abstract

Coronavirus disease 2019 (COVID-19) has been shown to impact multiple organs, even in instances where patients did not show any symptoms. In this case report, we detail a six-year-old male child presenting with focal seizures without an antecedent history of epilepsy. The child presented with twitching movements on the right side of the face involving the oral cavity. Non-contrast brain MRI showed meningoencephalitis. He was given antibiotics, antipyretics, and antiepileptic drugs (AEDs), but his clinical condition continued to deteriorate despite treatment. Oropharyngeal and nasopharyngeal swabs tested positive for COVID-19. Thus, treatment was initiated for COVID-19 encephalitis and seizures with intravenous immune globulin (IVIG) and steroids. Frequency of seizures decreased dramatically after steroids were initiated and remained infrequent during the five days of steroid therapy. After steroids were discontinued seizures returned but were shorter, less frequent and manageable with AEDs. The child was discharged on AEDs and was seizure-free at six months of follow-up. The following case report details the disease and treatment pathway of the patient.

## Introduction

The coronavirus disease 2019 (COVID-19) pandemic has profoundly impacted the health of the global population. Presently, the World Health Organization reports that 255 million individuals have been infected with the disease, with over five million resultant deaths [1]. Initially, it was believed that COVID-19 only had a detrimental impact on the respiratory system but, as the pandemic grew, it became clear that the condition was multisystemic and could have a detrimental impact on multiple organs [2,3]. Less commonly, COVID-19 is also known to cause neurological symptoms, with some studies reporting neurological symptoms in 30% of those infected, with higher prevalence among those with severe disease [4,5]. The most commonly reported neurologic symptoms include spasticity, movement disorders, seizures, headaches, altered taste, anosmia, dizziness, and cerebrovascular incidents [6,7]. In addition, de-novo focal seizures, as a consequence of COVID-19, have been reported in a small number of case reports, but we did not find any that described them in detail in pediatric patients [8].

Within the current case report, we describe a six-year-old previously healthy male child, who developed focal seizures that appeared to be associated with COVID-19. By reporting this case, we intend to increase healthcare providers' knowledge in treating children with the disease. Specifically, we want to raise awareness of the potential for neurological complications to occur within this patient population.

## Case presentation

A six-year-old previously healthy male child presented to our tertiary care hospital from a remote local pediatrician's office with a history of fever (five days), cough (three days), and new onset of multiple abnormal involuntary movements. The parents described twitching movements on the right side of his face, with deviation of the mouth and drooling of saliva from the left angle of the mouth. These episodes were accompanied by slurred speech and lasted for two to three minutes on each occurrence. The parents reported no history of loss of consciousness, eye-rolling, abnormal movements of limbs, or bowel/bladder incontinence. On examination, the child was noted to be tachycardic (118 bpm) and tachypneic (34/min), with low oxygen saturations (80%) in room air. He was afebrile (98.6°F) and had normal blood pressure (100/60 mmHg). Respiratory examination on auscultation revealed left-sided crackles. Neurological examination revealed drowsiness, impaired immediate memory recall, and slurred speech, but he was oriented to place and person and had no other focal neurological deficits.

The child was admitted to the neuro-intensive care unit, and treatment was initiated. He was kept on nil per oral (NPO) and an orogastric (OG) tube was placed. After OG tube placement, oxygen was given via face mask at 5 L/min to maintain saturations above 95%. The patient was started on IV fluids (0.45% dextrose) at 2/3 normal requirement (in view of meningitis). Injection ceftriaxone IV 2g 12 hourly (200 mg/kg/day) and injection acyclovir IV 300 mg eight hourly (15 mg/kg/dose) were started because of high suspicion of herpes simplex encephalitis. Lorazepam, 1 mg IV, was ordered for acute seizure episodes. Additional supportive treatment included injection paracetamol 300 mg IV eight hourly (15 mg/kg/dose), injection pantoprazole 20 mg IV 24 hourly (1 mg/kg/day), and injection ondansetron 3 mg IV 12 hourly (0.15mg/kg/dose), as needed. Although lorazepam was effective for acute episodes, seizures continued to occur. Injection levetiracetam IV was added, beginning with a 400 mg loading dose (20 mg/kg/dose), followed by 200 mg IV given 12 hourly (10mg/kg/dose).

**Figure 1:**
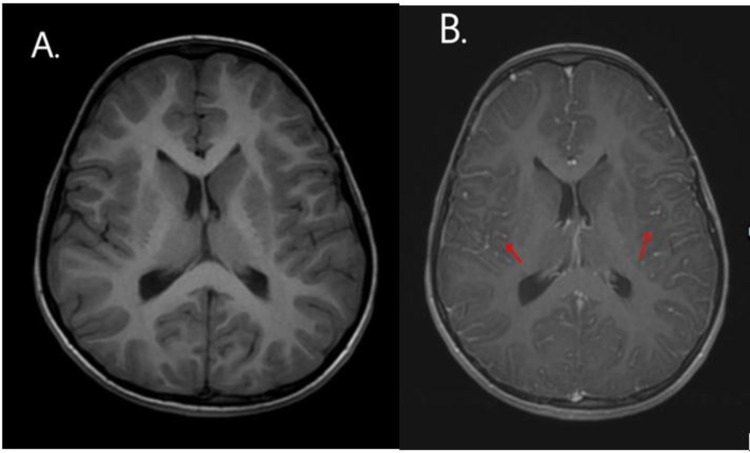
Axial T1-weighted MRI of a six-year-old boy with COVID-19 encephalopathy (A) without contrast - normal and (B) with contrast - diffuse leptomeningeal involvement in bilateral cerebral hemispheres COVID-19: coronavirus disease 2019

**Figure 2:**
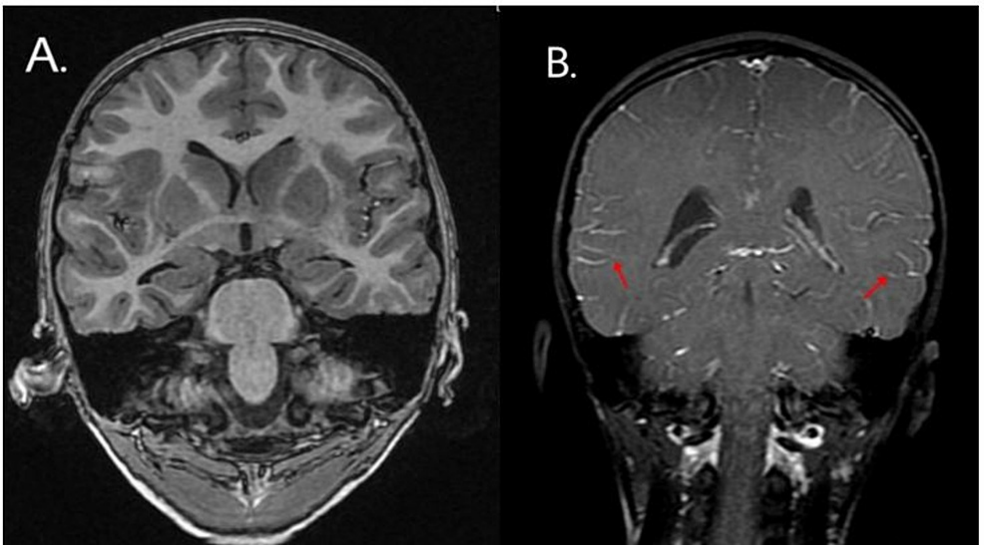
Coronal T1-weighted MRI of a six-year-old boy with COVID-19 encephalopathy (A) without contrast - normal and (B) with contrast - diffuse leptomeningeal involvement in bilateral cerebral hemispheres COVID-19: coronavirus disease 2019

**Figure 3:**
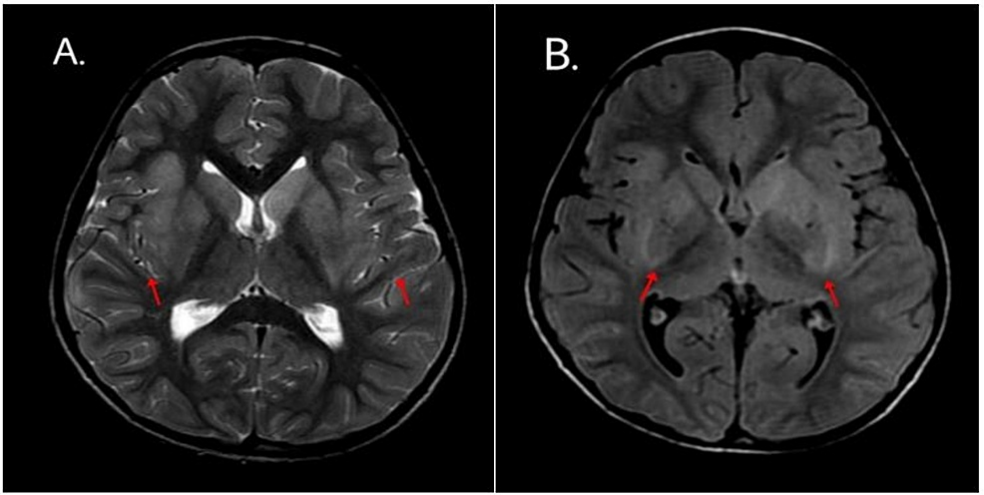
Axial (A) T2-weighted MRI and (B) FLAIR MRI of a six-year-old boy with COVID-19 encephalopathy show signs of symmetrical increase in signal intensity bilateral perisylvian cortex and bilateral basal ganglia suggestive of edema COVID-19: coronavirus disease 2019; FLAIR: fluid-attenuated inversion recovery

A complete blood count with differential (CBCD) showed an elevated total leukocyte count of 19,510 cells/mm^3^ with 78.5% neutrophils and 13.9% lymphocytes. C-reactive protein (CRP) was elevated at 53.6 mg/dL. A chest x-ray performed on admission revealed bilateral lung parenchymal involvement with right upper lobe infiltrates and left lower lobe consolidation. A CT lung screen showed a left basal lung collapse attributed to aspiration rather than infection. The MRI brain plain with contrast showed diffuse meningeal enhancement on both sides and altered signal intensity in the bilateral capsule-ganglionic and temporal regions - features suggestive of probable meningoencephalitis (Figures 1A-3B).

Cerebrospinal fluid (CSF), obtained after the MRI, was within normal limits showing no significant increase in WBC, and the viral meningitis panel (including herpes simplex virus polymerase chain reaction {HSV PCR}) was negative. Because the brain MRI showed symmetrical involvement, we ordered a CSF autoimmune encephalitis panel, including tests for serum neuromyelitis optica (NMO) and anti-myelin oligodendrocyte glycoprotein (anti-MOG) antibodies. The autoimmune panel returned all negative results. The negative CSF analysis suggested the encephalitis was an inflammatory reaction and not an acute viral leptomeningeal encephalitis. Oropharyngeal and nasopharyngeal swabs were taken to test (reverse transcription-polymerase chain reaction {RT-PCR}) for COVID-19 and indicated the patient was infected with the disease. However, D-dimer (523 ng/mL), serum ferritin (167 u/L), and serum lactate dehydrogenase (LDH) (196 U/L) were all within normal range, with interleukin-6 (IL-6) (8.17 pg/mL) being mildly elevated. Throughout the patient's stay in the hospital, CRP showed a decreasing trend.

A brief EEG was performed on day two of admission, after starting antiepileptic drugs (AEDs) and while the patient was stable and not having any seizure activity. No epileptiform activity was observed; however, based on the classic clinical presentation and the experience of the pediatric neurologist the abnormal movements were considered to have a high correlation with seizure activity. Later on day two, the seizures returned; therefore, lacosamide was added, with injection lacosamide IV 200 mg administered at a loading dose (10 mg/kg) followed by 100 mg 12 hourly (5 mg/kg/dose).

The child continued to have frequent, afebrile seizures, each lasting at least three to five minutes. Lorazepam continued to be effective for acute episodes (evidence that the involuntary movements represented seizure activity). However, because of the persistent seizures, up to five days, one lasting more than five minutes, on day three of admission, he was started on maintenance therapy with sodium valproate IV 200 mg 12 hourly (10 mg/kg/dose) and injection phenytoin IV 20 mg 12 hourly (2 mg/kg/day). Because blood culture, CSF culture, and HSV PCR were negative, acyclovir and ceftriaxone were discontinued. Despite the AEDs, the child continued to have focal seizures and a deteriorating sensorium. Changes in behavior, including increased sleepiness, decreased interaction with parents, and decreased eye contact, were also noted. Early on the fourth day of admission, he was started on injection methylprednisolone IV 600 mg 24 hourly (30 mg/kg/day), given for five days, and intravenous immunoglobulins IV 20 g (1g/kg/day), given for two days.

Following the administration of steroids, the frequency of seizure episodes decreased dramatically. The child’s symptoms improved, and, as a result, he was taken off oxygen support. His sensorium and behavioral changes improved, and he remained seizure-free during the whole course (five days) of methyl-prednisolone therapy. Three days after the completion of the five-day course of methylprednisolone, the child started having similar involuntary movements involving the right side of the face along with lips and tongue, though they were less frequent, only one to two episodes per day, and of shorter duration. As a result, the patient was additionally started on oral clobazam (5 mg in the morning and 2.5 mg at night). The seizure frequency and duration eventually decreased to an average of one every four to five days, each lasting less than five minutes. The patient was discharged at the end of the second week of admission.

The child was followed up for six months, with regular visits every two months. In the first three months of follow-up, the child had two episodes of seizures that were shorter in duration. The patient was advised to continue on oral AEDs, sodium valproate 200 mg twice daily, levetiracetam 400 mg twice daily, phenytoin 50 mg twice daily, and lacosamide 37.5 mg twice daily, for the next six months. Phenytoin was gradually tapered and stopped after one month, valproate and lacosamide were stopped after three months, and levetiracetam was to be continued for a year. Over the following months, there were no episodes of involuntary movements. Follow-up EEGs at three and six months did not show any seizure activity. The patient was advised to continue attending follow-up appointments for one year.

## Discussion

The current case report details a young male patient who presented with focal seizures and subsequently tested positive for COVID-19. To the best of our knowledge, this is the first such case reported in a pediatric population. We hope that by detailing his treatment regimen, this case can help to inform the treatment of such patients in the future.

As the pandemic swept across the globe, more detailed information has been gathered concerning the neurological impact of COVID-19 infection. Neurological signs related to infection are diverse and include, but are not limited to, anosmia, stroke, headaches, and dysgeusia [7]. There is also a growing body of evidence that suggests that these symptoms may persist long after acute COVID-19 infection has passed [9]. For example, a study published in 2021 from the United Kingdom found a range of neurological disorders within a relatively small cohort of children; disorders were diverse and included psychosis, behavior change, hallucinations, and peripheral nervous system involvement [10].

Overall, seizures appear to be a rare occurrence following COVID-19 infection [11]. At the same time, there is a limited but growing body of evidence that suggests that new-onset focal seizures and epilepsy should be considered a delayed central nervous system manifestation of COVID-19 infection [12]. Carroll et al. reported refractory status epilepticus in a 69-year-old African American woman six weeks after COVID-19 infection [13]. Previously published case reports have identified focal seizures in adult patients; for example, Vollono et al. reported its occurrence in a 78-year-old female patient [14]. Following a hospital admission, their patient was well enough to be discharged. A second case report details focal seizures in a 45-year-old patient, which, at the time of publication of the report, were still occurring and required ongoing medication [12].

At present, the mechanistic routes via which COVID-19 can negatively impact the nervous system are poorly understood [15]. The evidence is even more scarce among children, as the disease appears to be milder within this population when compared with adults, resulting in fewer long-term symptoms [16]. Proposed mechanisms include neurotropism, pro-inflammatory markers entering the nervous system, and the immune system's role following COVID-19 infection [17,18]. In addition, among children, multisystem inflammatory syndrome following COVID-19 infection may also play a causal role in developing post-viral seizures [19].

## Conclusions

Our case report details focal seizures following COVID-19 infection in a young child. The seizures were minimally responsive to AEDs but dramatically improved with the addition of a course of methylprednisone and intravenous immunoglobulins. After completion of steroids, the child's condition gradually improved on multiple antiseizure medications. The frequency of episodes declined from approximately five episodes a day on presentation to one every four to five days prior to discharge, and the child was seizure-free by six months of follow-up. Although focal seizures have been described in adults with COVID-19, they have not previously been reported in children infected with the virus. Further research, specifically data collection among young cohorts, is required to improve treatment and, subsequently, patient outcomes.
